# Lumbar Schwannoma as a Rare Cause of Radiculopathy in the Chiropractic Office: A Case Report

**DOI:** 10.7759/cureus.31758

**Published:** 2022-11-21

**Authors:** Eric C Chu, Robert J Trager, Wong J Yee, Kenny K Ng

**Affiliations:** 1 New York Chiropractic and Physiotherapy Centre, New York Medical Group, Kowloon, HKG; 2 Connor Whole Health, University Hospitals Cleveland Medical Center, Cleveland, USA; 3 College of Chiropractic, Logan University, Chesterfield, USA; 4 Oncology, reVIVE Oncology and Cancer Centre, Mong Kok, HKG

**Keywords:** neoplasms, low back pain, cauda equina, neurilemmoma, chiropractic

## Abstract

Cauda equina tumors are rare, slow-growing, and typically benign. These tumors present with low back pain resembling disc displacement with radiculopathy and thus may go undiagnosed for months.

A 52-year-old, otherwise healthy man presented to a chiropractor with a one-year history of worsening low back pain radiating to the right lower extremity, rated an 8/10 in severity and aggravated by recumbency. Previously, his primary care physician had ordered radiographs revealing mild lumbar degenerative changes, prescribed a non-steroidal anti-inflammatory medication, and referred him to an orthopedist and physical therapist. There had been no change in symptoms. Upon examination by the chiropractor, the patient had neurologic deficits, and due to progressive worsening, the chiropractor recommended magnetic resonance imaging (MRI), which the patient deferred due to cost. The chiropractor initiated a trial of care, with initial success; however, the patient’s symptoms recurred, and he consented to an MRI. MRI revealed an intradural extramedullary lumbar tumor, and the chiropractor referred the patient to an oncologist, who referred the patient to a neurosurgeon. The neurosurgeon surgically removed the mass, with a biopsy confirming a schwannoma. The patient had significantly improved six weeks after surgery.

This case highlights a patient with chronic low back pain for whom a chiropractor identified a cauda equina tumor and referred him for further evaluation and surgery. Clinicians should consider night pain and persistent symptoms, despite conservative care, as red flags warranting further investigation in those with low back pain. Providers should refer for neurosurgical evaluation when clinical and radiological findings suggest a cauda equina tumor.

## Introduction

Lumbosacral radiculopathy, also called sciatica, is a common condition involving low back pain radiating to the lower extremity with sensory, motor, and/or reflex deficit(s). While this condition is most commonly caused by lumbar disc herniation, it may rarely stem from more serious pathology, such as tumors [1]. Tumors of the cauda equina (the bundle of lumbar, sacral, and coccygeal nerves in the lumbar spine) are uncommon, typically benign, and slow-growing [2,3]. However, given their location, these tumors have the potential to cause symptoms typical of degenerative etiologies of low back pain and may go undiagnosed for several months [2]. Accordingly, it is important for providers who commonly manage lower back pain, such as chiropractors, to be aware of their clinical features and management.

Cauda equina tumors only affect 0.03 per 100,000 persons per year [2] and account for less than 1% of patients with lumbosacral radiculopathy [4]. The most common cauda equina tumors include (1) schwannomas and ependymomas, followed by (2) metastases [3,5]. Cauda equina tumors are diagnosed at a median age of 50 and have no sex predominance [5].

Tumors of the cauda equina typically present with low back pain, with or without motor or sensory symptoms, and occasionally bowel or bladder dysfunction [2,3,6]. Classically, symptoms are worsened with recumbency; however, this finding is only present in 58% of cases [7]. Pain related to an intraspinal tumor may also arise after trauma or exertion [8]. Examination findings may resemble those of lumbar disc herniation, with neural tension signs and sensorimotor deficits [5,7,9].

There is often a delay in the diagnosis of cauda equina tumors, possibly due to their slow-growing nature [2,3], initial lack of neurological signs [5], and/or resemblance of clinical features to lumbar disc herniation [5,7,9]. In one study (n=28), the mean time to diagnosis was 22 months [5]. Unfortunately, by the time these tumors are found, they are often large [3].

Despite the challenges in diagnosis, early identification of cauda equina tumors is important. Any diagnostic delay may allow irreversible neurologic deficits [6,10]. While primary cauda equina tumors are usually benign, they may, very rarely, undergo malignant transformation [11]. These tumors may be acutely exacerbated by trauma or may cause a spontaneous hemorrhage [2,3]. Accordingly, spinal manipulative therapy, a common treatment utilized by chiropractors, is contraindicated in such patients [12].

Considering that cauda equina tumors are a rare but potentially serious cause of low back pain that may go undiagnosed, we present a case in which a chiropractor identified a cauda equina tumor and referred the patient for further evaluation. The patient ultimately improved with surgery.

## Case presentation

A 52-year-old otherwise healthy man presented to a chiropractor with a one-year history of low back pain that worsened over the past 10 weeks to include radiation into the right buttocks and knee and a sensation of burning and numbness in the posterior right thigh, calf, and dorsum of the foot. The symptoms began insidiously, and he denied any history of trauma. The symptoms were alleviated by walking, which he did frequently as a maintenance worker, and were aggravated by lying in bed and prolonged sitting. He noted that he only slept for two hours per night due to pain. He denied bowel and bladder disturbances, a specific injury, previous surgery, spinal injections, or falls. Further, he denied taking medication. The patient was a social drinker, a non-smoker, and had no family history of cancer or spinal disorders. His World Health Organization Quality of Life Score was 68%.

**Figure 1:**
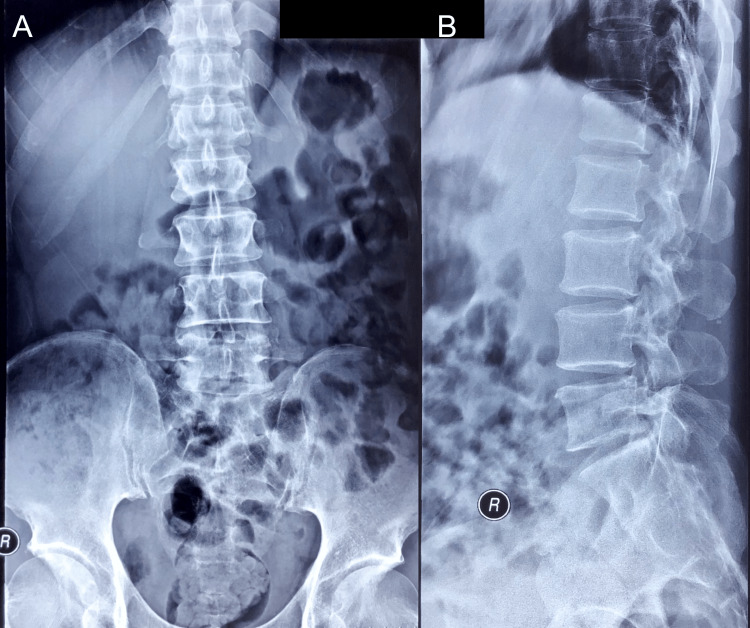
Lumbar spine radiographs taken prior to presentation to the chiropractor. The anteroposterior (A) and lateral (B) views appear normal. The radiology report noted “minimal degenerative findings.”

The patient initially visited his primary care provider when the low back pain began to worsen, two months before consulting with the chiropractor. The primary care provider ordered lumbar radiographs (Figure 1) and referred him to an orthopedist. The orthopedist provided a working diagnosis of lumbosacral radiculopathy related to lumbar spondylosis. This provider prescribed a nonsteroidal anti-inflammatory medication and referred the patient for physical therapy, which involved lumbar mobility and hip strengthening exercises, without further investigation. Since neither of these therapies provided the patient with relief, the patient presented to a chiropractor.

Upon examination, the chiropractor found the patient to have a normal lumbar range of motion; however, end-range flexion reproduced symptoms of pain and tightness in the right lower extremity. Straight-leg raising was painless on the left, but on the right, it reproduced a sensation of tightness in the right lower extremity. The sacroiliac compression test and prone extension test exacerbated local low back pain when performed on the right side. Valsalva and sacral thrust tests were negative. The patient’s knee ranges of motion were within normal limits, and a patellar compression test, a grinding test, and apprehension tests were negative. The sensitivity to light touch was intact. Strength testing was normal in the lower extremities except for knee extension and ankle dorsiflexion, which were graded 4/5 (on the Medical Research Council Scale).

The chiropractor’s differential diagnosis chiefly included discogenic radiculopathy of L5 and/or S1 given the patient’s signs and symptoms of nerve root involvement (e.g., strength deficits, neural tension). Considering the lumbar radiographs, which showed "minimal" degenerative changes, and the patient’s symptoms, which had worsened over the past 10 weeks despite conservative care, the chiropractor recommended a lumbar MRI at the first visit. However, the patient chose to avoid an MRI given its cost and preferred to initiate a trial of care instead.

The chiropractor began treatment with three visits per week over the course of three weeks. The chiropractor utilized lumbar flexion-distraction therapy, which involved a mechanically assisted joint mobilization technique including distraction and flexion-extension motions. The chiropractor also utilized pulsed electromagnetic field therapy (Hi-Power Magnetic Therapy, Bodycare®, Korea), a non-contact modality intended to alleviate pain that was focused on the low back for 15 minutes at each visit. The patient noted relief from his low back pain, which subsided to moderate severity (i.e., 4-6/10) over the first week, and noted improvements in sleep quality. During the second week of care, the patient’s pain further reduced to a mild level (i.e., 1-3/10). Given the patient’s improvements, the visit frequency was reduced to once per week after the third week of treatment.

However, during the 12th week of treatment, the patient’s pain relapsed without specific injury and again became severe and interrupted his sleep. Given this recurrence of symptoms, the chiropractor again recommended an MRI, which the patient consented to and obtained the following week.

**Figure 2:**
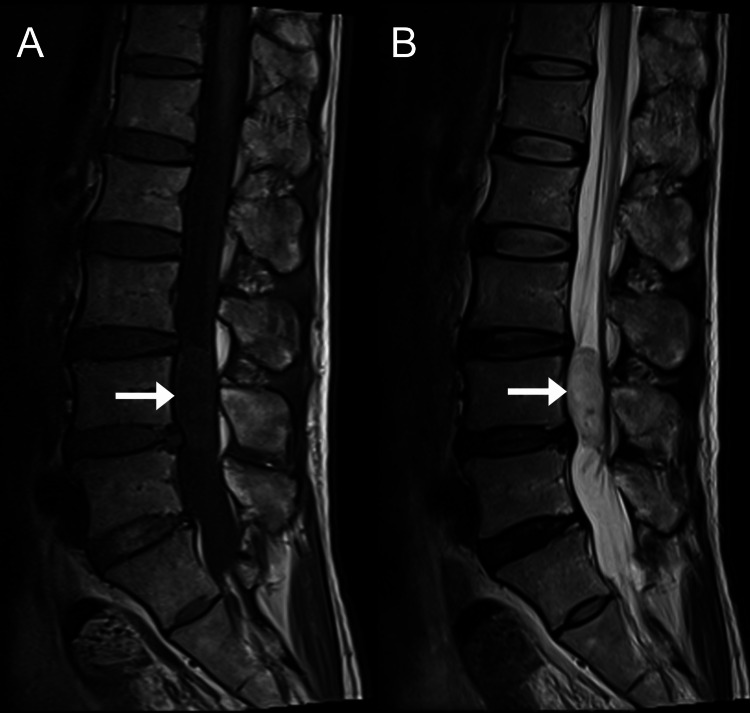
Lumbar spine magnetic resonance imaging, sagittal views. An intradural extramedullary mass is evident occupying the entire spinal canal at L4 level (arrows), measuring 1.98 cm × 1.32 cm × 3.73 cm, which is isointense on T1-weighted sequence (A), and slightly hyperintense on the T2-weighted sequence (B). Compression of the cauda equina is also evident.

**Figure 3:**
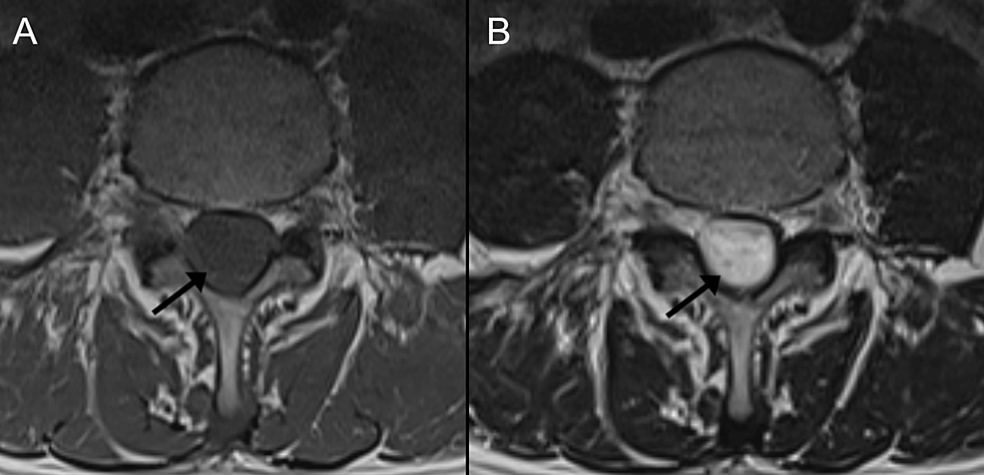
Lumbar spine magnetic resonance imaging, axial views at the level of L4. A mass occupies the entire spinal canal (arrows) which is isointense on the T1-weighted sequence (A), and slightly hyperintense on the T2-weighted sequence (B).

Thirteen weeks after the initial presentation to the chiropractor, the MRI, which was interpreted by a board-certified medical radiologist, suggested an intradural extramedullary mass compatible with a neuroma, a spinal meningioma, or other neoplasms (Figures 2-3). Also evident were mild circumferential disc bulges at L3/4, L4/5, and L5/S1, causing mild narrowing of the neural foramina at these levels.

After the tumor was identified via MRI, the chiropractor consulted with an oncologist within the same healthcare organization and scheduled the patient to see the oncologist via telemedicine the following day. The oncologist also saw the patient in person the following week and ordered computed tomography of the chest and abdomen to screen for additional masses. These tests were normal. The oncologist provided a working diagnosis of a benign intradural extramedullary tumor based on the lumbar imaging characteristics and absence of other findings and referred the patient to a neurosurgeon. In the interim, the oncologist cleared the patient to continue receiving chiropractic therapies, as these initially provided relief. However, the oncologist advised the chiropractor to only utilize low-force, gentle approaches. These included ongoing flexion distraction and pulsed electromagnetic field therapy, which were utilized weekly and provided the patient with transient relief.

Fourteen weeks after the presentation to the chiropractor, the patient saw a neurosurgeon, who evaluated the patient, demonstrating the weakness of dorsiflexion and the limitation of straight leg raising. The neurosurgeon advised surgery due to the large size of the lumbar mass, the presence of neurological deficits, and worsening despite conservative care.

Nineteen weeks after the initial presentation to the chiropractor, the patient underwent surgery. The neurosurgeon performed a laminectomy at L4 and completely excised the lumbar mass, and he subsequently sent the tumor specimen for histological examination. Macroscopically, the tumor was solid, tan/yellow, and 3.2 cm in length. Microscopic examination revealed spindle cells arranged in whorls and fascicles with focal palisading nuclei. There was no cellular pleomorphism, mitosis, or necrosis, and no evidence of malignancy. Based on these findings, the pathologist confirmed a diagnosis of schwannoma. Considering the surgery was successful and without complication, the neurosurgeon discharged the patient to recover at home with post-operative pain medication (tramadol).

Five weeks after surgery, the patient reported near-complete resolution of his symptoms, with the only remaining problem being mild, intermittent local low back pain. He provided written consent for the publication of this case and any accompanying images.

## Discussion

This case illustrates an adult man with progressive lumbosacral radicular symptoms that previous providers ascribed to degenerative lumbar findings, yet which had failed to respond to conservative treatment. A chiropractor identified a cauda equina tumor via MRI after an unsuccessful trial of care and referred the patient for further evaluation, who ultimately underwent surgery with a positive outcome.

In the current case, the patient had typical symptoms of radiculopathy, with a sensorimotor distribution most consistent with L5 and/or S1 nerve root lesion(s). One distinguishing finding was the relatively normal lumbar radiograph, which failed to explain the patient’s worsening symptoms [9]. In general, radiographs should be interpreted with caution as they have limited sensitivity to detect underlying cauda equina tumors and are frequently normal in such patients [5]. Another potentially important finding was an increase in symptoms with lying down, which is a classic symptom of cauda equina tumors, although it was not present in all cases [7].

It is challenging to explain why the patient initially obtained relief from the multimodal chiropractic treatments. It is possible that the patient’s coexisting degenerative lumbar changes contributed to his symptoms, however, these findings were mild and did not cause neural compression on MRI. Another possibility is that lumbar flexion-distraction therapy, which may temporarily widen the lumbar spinal canal, alleviates nerve compression related to the lumbar schwannoma [13]. Further, it is possible that the patient’s fluctuation in symptoms was unrelated altogether, related to the patient's expectations of chiropractic care, or a broad anti-nociceptive effect of the therapies provided [14]. Although the intent of the chiropractic treatment was not to treat the schwannoma itself, there were no adverse events in relation to the therapies provided.

Identification of a cauda equina tumor by a chiropractor is seldom reported, with only a few published cases, according to a literature search of Google Scholar, PubMed, and the Index to Chiropractic Literature on October 5, 2022 [13,15,16]. In one similar case of schwannoma, a 56-year-old woman presented with low back pain radiating to the L5 dermatome and weakness of ankle dorsiflexion, which was initially suspected to be lumbar disc herniation but later found to be schwannoma [15]. Another case described a 37-year-old woman with a four-year history of worsening back and leg pain, ultimately identified as an intradural schwannoma at T12-L2 [16]. A final case described a 45-year-old man with a myxopapillary ependymoma causing bilateral lumbar radiculopathy [13].

Several imaging guidelines consider the presence of red flags, signs of potential severe pathology, as indications to obtain a lumbar MRI for patients with low back pain [17]. As in the current case, symptom duration of four to six weeks without response to conservative care and night pain were both red flags warranting appropriate lumbar MRI [17]. While the diagnostic accuracy of individual red flags for low back pain is limited, red flags found in combination are potentially more useful [18].

Early diagnosis of a cauda equina tumor is paramount. Although these tumors are typically benign and slow-growing, delayed diagnosis may lead to permanent neurological deficits [6,10]. In addition, they may bleed after trauma, leading to acute cauda equina syndrome [2,3] and representing a contraindication to spinal manipulation [12]. Plain radiographs are only useful in select cases [19], and as illustrated in the current case, they may fail to detect any underlying cauda equina tumor. The gold standard imaging modality to detect cauda equina tumors is MRI; however, computed tomography, cerebrospinal fluid analysis, angiography, and electrodiagnostic testing may also be useful in diagnosis [19]. The mainstay of treatment for a cauda equina tumor is surgery, with many cases requiring laminectomy to allow access to the tumor [19].

## Conclusions

Cauda equina tumors may be easily overlooked by clinicians as they produce symptoms with a resemblance to more common musculoskeletal disorders and may not be detected by plain film radiography. Clinicians should investigate further with testing such as MRI when patients demonstrate red flags such as night pain or fail to improve with conservative treatment. Providers should refer patients for neurosurgical evaluation when testing is suggestive of a cauda equina tumor.
